# Sudden Death with Nervous Phenomena in Case of Wound of Lung

**Published:** 1918-09

**Authors:** 


**Sudden Death With Nervous Phenomena in Case of Wound of Lung.** Piery, Lyon Med., April, 1917, reports the case of a soldier wounded by a ball entering at the base of the neck, the wound of exit of the size of a dime, being between midway between the posterior border of the scapula and the spine. Signs of haemothorax and pneumothorax were found 10 days later but not at X-ray examination 6 days later still. At this time, the patient had rapid respiration but there was no cyanosis and the pulse was normal. He soon lost consciousness and oxygen, ether and caffeine injections failed to revive him, death occurring in half an hour. A fracture of the 4th rib was demonstrated but no explanation of the sudden death.

